# Sex-Specific Effects of Obesity Severity on Circulating Inflammatory Mediators and Immune Cell Gene Expression

**DOI:** 10.3390/ijms27073314

**Published:** 2026-04-07

**Authors:** Xavier Capó, Miguel David Ferrer, Antoni Sureda, Maria Magdalena Quetglas-Llabrés, Margalida Monserrat-Mesquida, Maria del Mar Bibiloni, Silvia García, Marina Ródenas-Munar, Lucia Ugarriza, David Mateos, Cristina Bouzas, Josep A. Tur, Antoni Pons

**Affiliations:** 1 Research Group in Community Nutrition and Oxidative Stress (NUCOX), University of Balearic Islands, 07122 Palma de Mallorca, Spainantoni.sureda@uib.es (A.S.); margalida.monserrat@uib.es (M.M.-M.); m.rodenas@uib.cat (M.R.-M.); pep.tur@uib.es (J.A.T.); 2 Translational Research in Aging and Longevity (TRIAL) Group, Health Research Institute of the Balearic Islands (IdISBa), 07120 Palma de Mallorca, Spain; 3 Laboratory of Physical Activity Sciences, University of Balearic Islands, 07122 Palma de Mallorca, Spain; 4 Renal Lithiasis and Pathological Calcification Group (LiRCaP), Research Institute of Health Sciences (IUNICS), University of the Balearic Islands, 07122 Palma de Mallorca, Spain; miguel-david.ferrer@uib.es; 5 Health Research Institute of the Balearic Islands (IdISBa), 07120 Palma de Mallorca, Spain; 6 CIBEROBN (Physiopathology of Obesity and Nutrition), Instituto de Salud Carlos III, 28029 Madrid, Spain

**Keywords:** obesity, inflammation, cytokines, adipokines, immune cells, mitochondria

## Abstract

Obesity is characterized by chronic low-grade inflammation and immunometabolic dysfunction. However, the influence of obesity degree on circulating inflammatory mediators and immune-cell–specific molecular pathways remains poorly defined. We aimed to examine the effects of obesity severity on plasma cytokines and adipokines, and on the expression of inflammatory, antioxidant, and mitochondrial genes in peripheral immune cells, with emphasis on gender differences. In this cross-sectional study, 134 adults aged 55–80 years were stratified into overweight, moderate, or severe obesity groups. Plasma cytokine and adipokine levels were quantified with multiplex immunoassays. Gene expression in peripheral blood mononuclear cells and neutrophils was measured by quantitative PCR. Standard hematological parameters were assessed. Two-way analysis of variance and correlation analyses were performed to evaluate associations between obesity severity, gender, circulating biomarkers, and gene expression. Severity of obesity was associated with distinct alterations in circulating inflammatory profiles in a gender-dependent manner. Women exhibited progressive increases in plasma interleukin (IL)-6 with obesity severity, whereas men with severe obesity demonstrated elevated IL-15 and IL-1rα plasma levels. Hematological responses also differed by gender. In contrast, expression of classical inflammatory genes in immune cells was largely unchanged across obesity categories. Women showed higher expression of antioxidant and mitochondrial genes than men, suggesting gender-specific resilience in redox and mitochondrial pathways. Correlations between circulating inflammatory mediators and immune-cell gene expression were generally weak. Obesity severity and gender modulate systemic inflammatory, while immune-cell transcriptional responses are limited, highlighting the importance of gender-specific immunometabolic characterization in obesity research.

## 1. Introduction

Obesity is a complex, multifactorial condition characterized by excessive accumulation of fat in adipose tissue and ectopic lipid deposition in non-adipose organs [1]. A sedentary and unhealthy lifestyle, overnutrition and chronic positive energy balance lead to triglyceride accumulation and ectopic lipid storage, and are related to several obesity-associated co-morbidities. The sustained energy imbalance is accompanied by chronic low-grade inflammation and adipocyte dysfunction, leading to insulin resistance and further ectopic lipid deposition [2]. White adipose tissue remodelling, through changes in expansion mode, microenvironment, cellular phenotype, and fat distribution, contributes to the development of metabolically healthy versus unhealthy obesity [3]. Importantly, sex-specific differences in adipose tissue distribution and ectopic fat deposition have been described, which may underlie divergent metabolic and inflammatory profiles between men and women [4]. Obesity is associated with insulin resistance and several co-morbidities associated with metabolic syndrome such as type 2 diabetes, cardiovascular diseases, non-alcoholic fatty liver, inflammatory-based diseases such as psoriasis, infertility, and some cancers [5,6]. The progression from overweight to moderate, severe and morbid obesity increases the incidence of these associated co-morbidities [7,8]. Chronic low-grade inflammation is a central and unifying mechanism in obesity pathogenesis, characterized by the early activation of inflammatory programs during adipose tissue expansion and their persistence over time, which permanently biases the immune system toward a proinflammatory phenotype [9]. This chronic inflammatory state triggers the activation and infiltration of pro-inflammatory immune cells and a dysregulated production of high levels of pro-inflammatory cytokines [10].

White adipose tissue secretes a wide range of adipokines, including cytokines and chemokines such as tumour necrosis factor-α (TNFα), interleukin-6 (IL6), interleukin-15 (IL-15), and monocyte chemoattractant protein-1 (MCP1), which play a key role in the regulation of metabolism and immune function. In addition, adipose tissue produces hormone-like factors such as leptin, adiponectin, resistin, and osteoprotegerin (OPG), which exert autocrine effects on adipose tissue itself and paracrine and endocrine actions on neighbouring and distant organs [11,12]. Through these mediators, adipokines signal to multiple organs, including the brain, liver, pancreas, kidney, skeletal muscle, and immune system, thereby regulating energy balance, appetite, inflammation, blood pressure, and body weight [10].

Leptin is a central regulator of energy homeostasis, primarily by inhibiting hunger, but it also exerts potent immunomodulatory effects. It promotes CD4 T-cell activation leading to pro-inflammatory cytokine secretion (TNFα, IL6, IL12), up regulates activation markers on monocytes (CD11b, CD11c, MHC class II, CD25, CD38, CD69), and stimulates reactive oxygen species (ROS) production as well as polymorphonuclear cell chemotaxis [10]. Circulating plasma leptin levels are mainly influenced by gender, metabolic hormones, and total body mass index (BMI) [13]. However, in obesity, chronically elevated leptin levels are frequently accompanied by central leptin resistance, particularly at the hypothalamic level, which blunts its anorexigenic effects while preserving its pro-inflammatory actions, thereby contributing to metabolic and immune dysregulation [14].

Osteoprotegerin (OPG), a member of the tumour necrosis factor superfamily, is produced not only by bone but also by adipose tissue. It plays a regulatory role in mediating adipogenesis during obesity development and may be involved in the inflammatory process associated with obesity [15]. In this sense, obesity has been associated with reduced circulating OPG levels, probably reflecting a compensatory mechanism, while elevated plasma OPG levels are considered indicative of an increased risk of metabolic dysfunction [16]. Ghrelin, a well-known gut hormone, is a key regulator of appetite and body weight, as it increases the hunger sensation and food intake [17]. Resistin, produced mainly by macrophages and adipose tissue, has proinflammatory actions and may link obesity to insulin resistance [18]. Binding of resistin to Toll-like receptor 4 (TLR-4) promotes the activation of the pro-inflammatory cytokines IL-6, IL-1β, and TNFα and induces the upregulation of several adhesion molecules in vascular endothelial cells through NFκB signalling [9]. IL-15, which is also produced by adipose tissue, displays a dual role by exerting both pro- and anti-inflammatory effects. IL-15 has been shown to protect against type 2 diabetes and to reduce body weight gain without suppressing food intake. Moreover, lL15 also is able to reduce weight gain without inhibiting food intake, reduce lipogenesis, stimulate brown fat function, enhance energy expenditure, and improve insulin sensitivity [10]. Collectively, adipokines and cytokines such as IL-6, TNF-α, and IL-1β secreted by adipose tissue act on immune cells, initiating local inflammation that can subsequently progress to a systemic inflammatory state. Adipose tissue inflammation is mediated through several interconnected signaling pathways, including the TLR-4/phosphatidylinositol-3′-kinase (PI3K)/Protein kinase B (Akt) signalling pathway, endoplasmic reticulum (ER) stress-derived unfolded protein response (UPR), and the inhibitor of nuclear factor kappa-B kinase beta (IKKβ)-NFκB pathway [19].

In addition to inflammatory mediators, adipose tissue and immune cells are closely linked to redox homeostasis. Oestrogens have been shown to enhance antioxidant capacity by upregulating the expression of antioxidant enzymes and modulating mitochondrial function [20,21]. Moreover, adipose tissue acts as an important site of oestrogen synthesis through aromatase activity, particularly in obesity, which may further influence inflammatory and oxidative stress responses in a sex-dependent manner [22].

The influence of obesity severity on the ability of peripheral immune cells to express antioxidant, inflammatory, and anti-inflammatory genes will contribute to understanding the role of these cells in maintaining low-grade inflammatory status associated with obesity. Key antioxidant mediators include superoxide dismutases (SOD1, SOD2), catalase (CAT), glutathione peroxidase (GPX), and nuclear factor erythroid 2–related factor 2 (NRF2), whose genes are located on different chromosomes and whose transcription is regulated by redox-sensitive nuclear receptors and transcription factors. These pathways can be modulated by metabolic status, hormonal milieu, inflammatory cytokines, and adipokines, highlighting the complex regulation of antioxidant defence mechanisms in obesity.

Mitochondria play a critical role in the differentiation, activation, and regulation of the immune cell response, in addition to their well-established function in energy production [23]. Upon activation, pro-inflammatory immune cells exhibit increased energetic demands to sustain processes such as cytokine synthesis, migration, and effector functions. Consequently, alterations in mitochondrial dynamics such as fission/fusion processes produce mitochondrial dysfunction, emerging as an important regulator of obesity complications [24]. Mitochondria, through the production of ROS acting as secondary messengers, are able to activate immune cells [25]. Dysregulation of mitochondrial dynamics is typically reflected by altered expression of fusion and fission proteins, leading to excessive mitochondrial fragmentation, increased ROS production, and reduced ATP synthesis [24]. These mitochondrial alterations may therefore link metabolic stress to impaired immune cell function and sustained inflammatory responses in obesity.

Therefore, the main aim of this study was to evaluate, according to obesity severity, plasma levels of key cytokines and adipokines and their association with the expression of inflammatory, antioxidant, and mitochondrial dynamics–related genes in peripheral blood mononuclear cells and neutrophils.

## 2. Results

The study participants were grouped by BMI into in three categories: overweight, moderate, and severe obesity, and results are presented separately for women and men. Anthropometric and clinical characteristics are presented in Table 1. Women presented significantly higher total cholesterol, high-density lipoprotein cholesterol (HDL-c) and low-density lipoproteins cholesterol (LDL-c) than men, particularly in the severe obesity group. Notably, fasting glucose levels were comparable across obesity severity groups, suggesting similar metabolic control among participants. Detailed statistical parameters, including degrees of freedom and F-values, are reported in the Table S1.

Table 2 presents effects of obesity degree on hematological parameters. Obesity severity significantly affected the hematocrit and the mean corpuscular volume (MCV) of erythrocytes. Severe obesity condition is associated with significantly lower hematocrit and MCV than overweight or moderate obesity in women, but not in men. Leukocyte and neutrophil counts were higher in overweight women than in overweight men and were also higher in overweight women compared with women with moderate or severe obesity, whereas men maintained similar counts across obesity categories. Lymphocyte counts were similar across groups for both genders, although women with severe obesity had higher lymphocyte counts than men with severe obesity. The neutrophil-to-lymphocyte ratio (NLR) was influenced by gender and obesity severity, since women with severe obesity had significantly lower NLR values than men with severe obesity. Eosinophils, basophils and platelet counts differed by gender; however, only eosinophil counts were significantly affected by obesity severity. Men tended to show decreased eosinophils with increasing obesity while women showed the opposite trend. The systemic immune-inflammatory index (SII) was influenced by obesity severity, with men in the moderate-obesity group showing the lowest SII. Detailed statistical parameters, including degrees of freedom and F-values, are reported in the Table S2.

The influence of obesity severity and gender on the interleukin and chemokine plasma levels are given in Table 3. Plasma IL-6, IL-15 and IL-1rα, as well as ghrelin were significantly influenced by the obesity severity and/or gender; IL-6 and ghrelin showed an interaction between these two factors. Women in the overweight condition presented significantly lower ghrelin levels than men in the overweight condition; ghrelin plasma levels tended to increase with obesity severity in women but decreased in men. IL-6 plasma levels tended to increase with obesity in women while remaining relatively stable in men; in severe obesity conditions, IL-6 concentration in women doubled those in men. IL-15 plasma levels maintained similar values in women across obesity categories but increased markedly with obesity in men; men with severe obesity had IL-15 plasma levels approximately three times higher compared with the overweight group and with women in the severe obesity group. Similarity, IL-1rα tended to rise with obesity and was lower in women than in men, with men in the severe obesity group showing the highest IL-1rα levels. Other analytes such as TNFα, gamma interferon (IFN-γ), IL-10, resistin, and leptin did not show consistent changes across obesity degrees. Detailed statistical parameters, including degrees of freedom and F-values, are re-ported in the Table S3.

**Figure 1:**
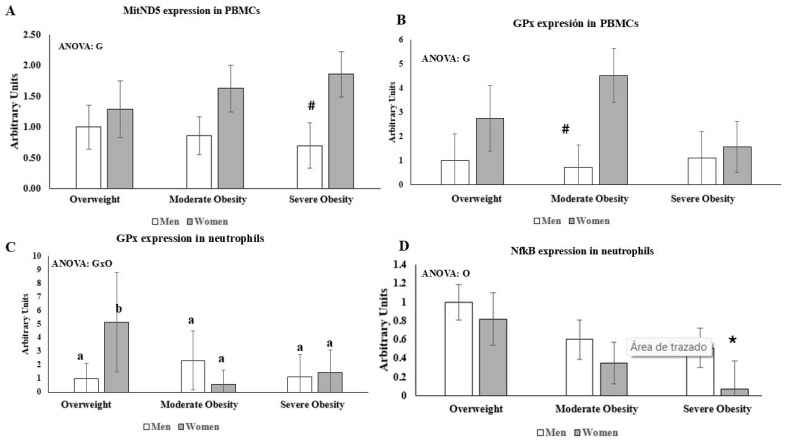
Effects of gender and obesity degree on gene expression. (**A**) MitND5 expression in PBMCs; (**B**) GPx expression in PBMCs; (**C**) GPx expression in neutrophils; (**D**) NFkB expression in neutrophils. Results are the mean ± SEM. Statistical analysis: Two-way ANOVA, *p* < 0.05.G, means significant differences due to gender; O means significant differences between different obesity degrees; GxO, means interaction between two factors. # Indicates significant differences male and female; * indicates differences respect overweight; When interaction exists between different statistical factors, different letters reveal significant differences.

Gene expression of inflammatory, antioxidant, and mitochondrial turnover markers in peripheral blood mononuclear cells (PBMCs) and neutrophils from men and women with different obesity severities is shown in Table S4 and Figure 1. No effects of gender or obesity degree were observed in TNFα, IL-1β, IL-10, toll like receptor (TLR)-2, TLR-4, cytochrome c oxidase subunit IV (COXIV), mitofusin (MTF)-1, MTF-2, NFκB, or cyclooxygenase 2 (COX-2) in PBMCs gene expression (Table S1). Similarity, no effects of gender or obesity degree were observed in TNFα, IL-1β, IL-10, TLR-2, TLR-4, COXIV, MTF-1, or COX-2 in neutrophils gene expression (Table S1). Figure 1 shows the genes were significantly influenced by gender or by the severity obesity in PBMCs and neutrophils. Gender significantly influenced the gene expression of both mitochondrial NADH dehydrogenase subunit 5 (MitND5) and glutathione peroxidase (GPx) in PBMCs (Figure 1A,B). PBMCs of women in severe obesity conditions presented significantly higher MitND5 expression than PBMCs of men in severe obesity conditions. An interaction between the gender and the obesity severity in GPx expression was observed in neutrophils (Figure 1C). Women in overweight conditions had significantly higher neutrophils GPx expression than overweight men and also than both moderate and severe obesity women. Neutrophil NFκB expression (Figure 1D) in women with severe obesity was significantly lower than this expression in overweight women. Detailed statistical parameters, including degrees of freedom and F-values, are re-ported in the Table S5.

**Figure 2:**
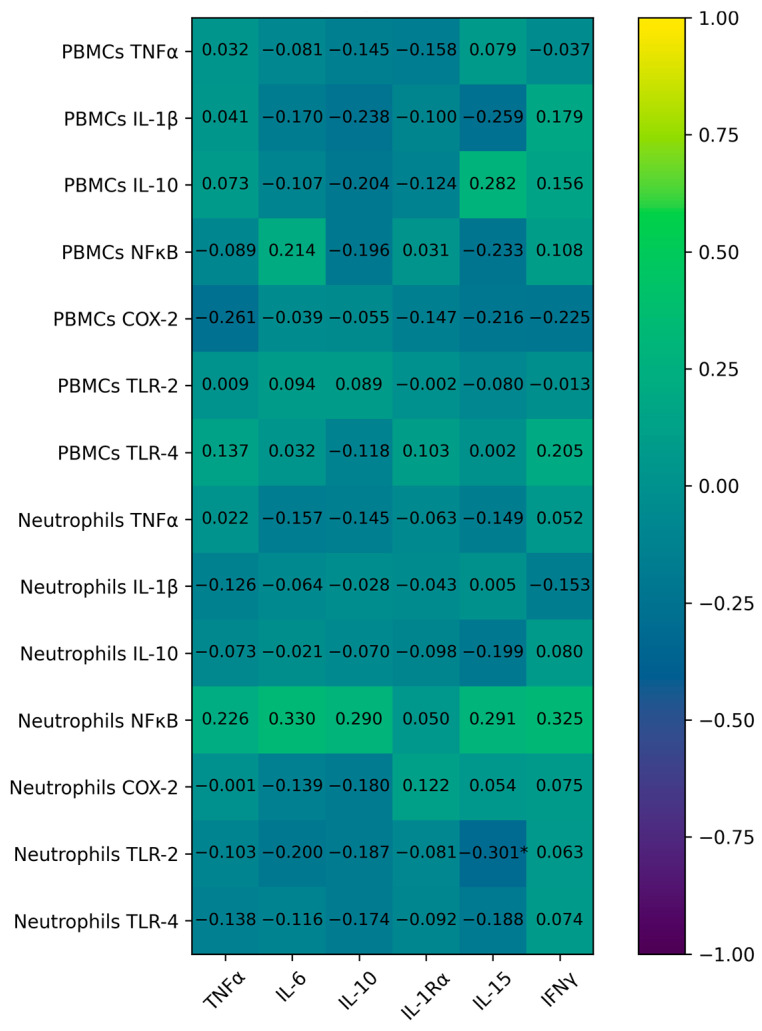
Correlation between PBMCs and neutrophil inflammatory genes and the cytokine plasma levels. Values are expressed as Pearson coefficient * Statistically significant *p* < 0.05; * *p* < 0.05.

Correlations between plasma cytokines and immune-cell gene expression in both PBMCs are presented in the Figure 2. Overall, plasma cytokine concentrations showed weak correlations with inflammatory-gene expression in PBMCs and neutrophils; the exception was a significant negative correlation between IL-15 plasma levels and neutrophil TLR-2 expression. On the contrary, cytokine plasma levels showed a strong significant correlation between their plasma values. The TNFα plasma levels were significantly correlated with the plasma levels of IL-15, IFN-γ, IL-6, and IL-10. The IL-6 plasma levels also were significantly correlated with IL-10, IL-15, and IFN-γ plasma levels. In addition, plasma levels of IL-15 were also significantly correlated with IL-10 and IFN-γ. The plasma levels of IL-1rα were only significantly correlated with the plasma levels of IFN-γ. Furthermore, the inflammatory genes expression in PBMCs was poorly correlated with the inflammatory genes expression in neutrophils; the neutrophil’s TNFα expression was significantly positively correlated with the PBMC’s COX-2 expression; the neutrophil’s TLR-2 expression was positively significantly correlated with PBMC’s IL-1β expression.

**Figure 3:**
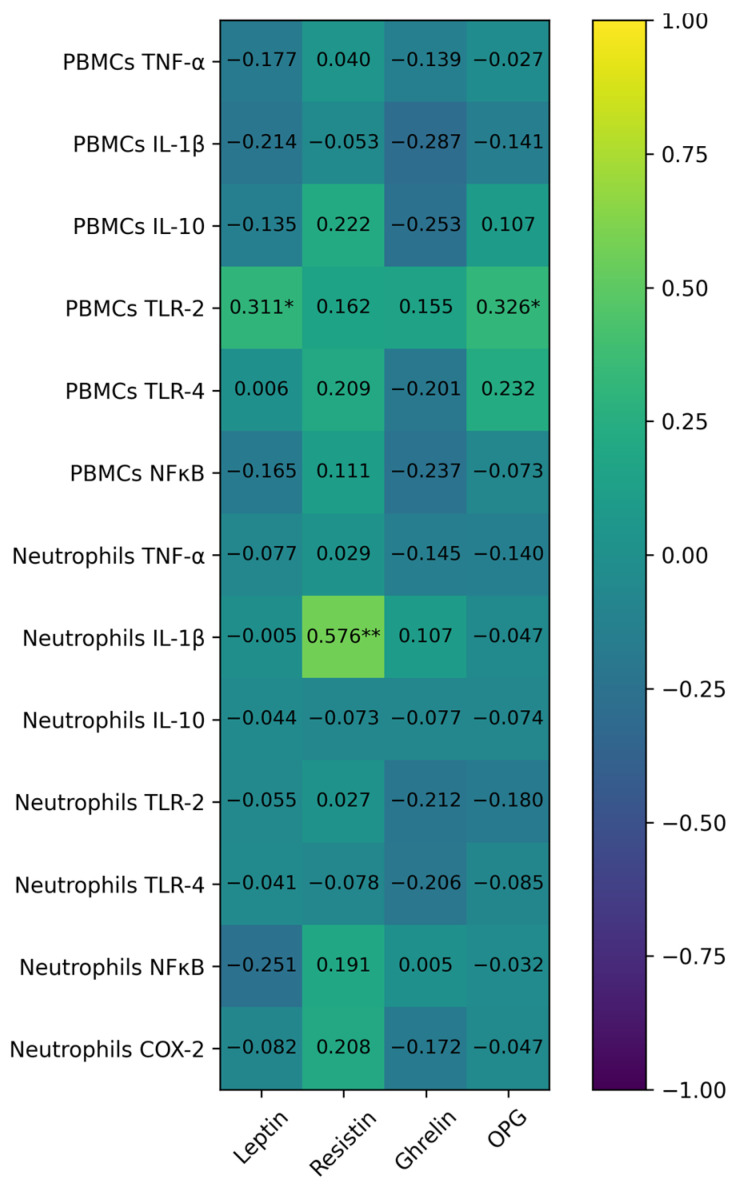
Correlation between PBMCs and neutrophil, inflammatory and the adipokines plasma levels. * *p* < 0.05; ** *p* < 0.01.

The correlation between PBMCs and neutrophil inflammatory genes and adipokine plasma levels are presented in the Figure 3. The TLR-2 gene expression in PBMCs correlated positively with both Leptin and OPG plasma levels, while no correlation between other plasma adipokines levels and inflammatory genes expression in PBMCs were evidenced. In the case of neutrophils, only IL-1β expression correlated with resistin plasma levels, while no correlation between other adipokine plasma levels and inflammatory gene expressions in neutrophils were observed. The Leptin plasma levels significantly correlated with the ghrelin plasma levels, and the resistin plasma levels significantly correlated with the OPG plasma levels.

**Figure 4:**
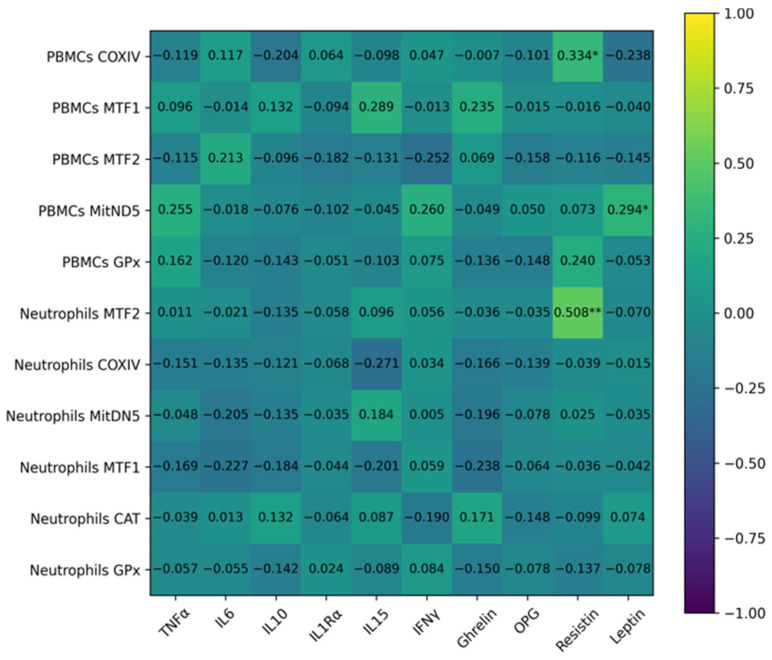
Correlation between PBMCs and neutrophil antioxidant and mitochondrial turnover genes, and the cytokine and adipokine plasma levels. R values express the Pearson correlation coefficient; *p* values express probability; * Statistically significant *p* < 0.05. * *p* < 0.05; ** *p* < 0.01.

Regarding mitochondrial and antioxidant genes (Figure 4), PBMC COXIV correlated positively with resistin, and PBMC MitND5 correlated with leptin. In neutrophils, a strong positive correlation was observed exclusively between resistin plasma levels and the expression of MTF-2.

## 3. Discussion

This study evaluated gender-specific patterns and the impact of increasing obesity severity on systemic inflammatory mediators, hematological parameters, and the expression of inflammatory, antioxidant, and mitochondrial-related genes in PBMCs and neutrophils. Rather than restating the study objective, the discussion focuses on the interpretation of the main findings and their biological and clinical implications. Overall, the results suggest that obesity severity and gender are associated with differential systemic inflammatory and hematological responses, whereas immune-cell gene expression changes appear more limited and context-dependent.

Hematological parameters were significantly influenced by both obesity degree and gender. Women presented greater variability across obesity categories, with severe obesity being associated with reduced hematocrit and MCV, whereas these indices remained stable in men. These findings are consistent with previous studies reporting changes in erythrocyte indices associated with obesity-related disturbances in iron metabolism [26,27,28]. Leukocyte and neutrophil counts were also more responsive to obesity severity in women, showing higher values in overweight women than in moderate obesity and in severe obesity, while men maintained relatively constant counts across all categories. Similar patterns were previously described in adults with metabolically healthy and unhealthy overweight or obesity [29], as well as in obese individuals without metabolic syndrome [30], although gender-specific differences were not addressed in those studies. However, given the heterogeneity of the groups and the inclusion of participants with obesity-related comorbidities, these findings should be interpreted with caution, as metabolic status and pharmacological treatments may have contributed to the observed variability.

The NLR is a widely used marker of systemic inflammation, which tends to increase with the degree of obesity and is also influenced by the gender [31]. In line with this, our results showed that NLR was influenced by both gender and obesity severity, increasing significantly only in men with severe obesity, while women exhibited a more attenuated response. This gender-dependent pattern may reflect differences in fat distribution and immune cell infiltration, as females typically display greater subcutaneous adipose tissue immune activity, whereas males show more pronounced visceral fat-associated inflammation [32]. Nonetheless, age, hormonal status, and metabolic health may all have an impact on the slight variations in NLR between the genders [33]. These factors may partly explain the observed gender-specific differences in NLR and cannot be excluded as contributors to the current findings.

Eosinophil counts followed opposite trends between genders, decreasing with obesity progression in men but increasing in women. Collectively, these findings suggest that the hematological adaptations to increasing adiposity are highly gender-dependent, with women exhibiting more pronounced fluctuations in erythrocyte and leukocyte indices, and men showing a clearer pro-inflammatory shift in cell-based markers such as NLR. These divergent patterns highlight the importance of incorporating gender stratification when interpreting hematological biomarkers in the context of obesity-related inflammation. Nonetheless, the potential influence of comorbidities, medication use, and unequal group sizes limits definitive conclusions, underscoring the importance of cautious interpretation.

Contrary to expectations, obesity severity did not uniformly increase classical pro-inflammatory cytokine plasma levels. For example, TNF-α, IFN-γ, IL-10, and resistin remained largely unchanged across obesity categories, suggesting that chronic low-grade inflammation in obesity does not necessarily manifest as linear increases in all circulating pro-inflammatory cytokines. It is important to emphasize that many participants with obesity were under pharmacological treatment, including antidiabetic and lipid-lowering therapies with known anti-inflammatory properties, which may have attenuated circulating cytokine levels. This pharmacological modulation likely contributed to the absence of progressive increases in these mediators and must be considered when interpreting these results. These results contrast with previous studies reporting increased levels of TNF-α, IFN-γ, and resistin plasma levels in patients with obesity [34]. A possible explanation is that the present cohort consisted exclusively of individuals with overweight or obesity, a context in which chronic low-grade inflammation is already established. Under these conditions, pro-inflammatory cytokines often reach a biological “*plateau*” where their circulating levels become relatively stable and no longer reflect incremental increases in adiposity [35,36]. However, as with other inflammatory markers, the potential influence of comorbidities and pharmacological treatments cannot be excluded.

In contrast, the marked gender-specific differences observed for IL-6, IL-1rα, and IL-15 responses to increasing adiposity support and expand upon earlier findings of gender differences in obesity-related inflammation. In the case of IL-6, women presented higher plasma IL-6 concentrations with obesity severity, reaching levels more than double those of men in severe obesity. These results are in accordance with previous evidence showing that women often exhibit a more reactive inflammatory milieu, especially for IL-6 [36,37]. This could be related to differences in adipose tissue distribution and remodeling [37].

IL-15 and IL-1rα showed a distinct pattern, with significant increases observed predominantly in men with severe obesity, suggesting a distinct inflammatory trajectory compared to women. In this sense, although IL-15 has frequently been reported to be negatively associated with BMI and fat mass [38,39], our data suggest a different behavior in the context of advanced obesity, particularly in men. In advanced obesity, particularly in men, circulating IL-15 may reflect a shift from muscle-derived to visceral adipose-tissue–derived IL-15, as visceral fat expansion and its stromal–immune compartment have been shown to express increased IL-15 in obesity. This could explain the divergence from previously reported inverse associations with BMI and may represent a compensatory metabolic response [40]. Similarity, elevated IL-1rα plasma levels observed in men with severity obesity are consistent with previous studies linking visceral adiposity to increased production of IL-1–related mediators, including IL-1rα by adipose tissue macrophages [41,42].

Ghrelin was the only adipokine which is significantly altered in our study, showing a clear gender-dependent pattern. Concretely, ghrelin concentrations tended to rise with obesity in women but declined in men. This divergent regulation has been previously described and is likely influenced by sex hormones and differences in energy homeostasis regulation between men and women [43,44].

Despite the clear alterations observed in circulating inflammatory mediators, the stability of gene expression profiles in immune cells across obesity categories suggests a more complex and compartmentalized inflammatory response than traditionally assumed. Key inflammatory genes, TNF-α, IL-1β, IL-10, TLR-2, TLR-4, and COX-2, as well as several mitochondrial- and oxidative-stress-related genes, were not significantly affected by obesity severity. This apparent dissociation between systemic inflammatory markers and immune-cell gene expression has been described previously. In this sense, it is evidenced that chronic metabolic inflammation is often originated in metabolic tissues such as adipose tissue, rather than circulating leukocytes. Adipose tissue macrophages and stromal cells are known to be primary contributors to obesity-associated inflammatory cytokines such as TNF-α, IL-6, and MCP-1 [45,46].

Several mechanisms may explain this dissociation. Chronic obesity may induce immune adaptation, reducing TLR signaling and cytokine production after prolonged metabolic stress [47]. In addition, the advanced age of the cohort likely contributes through immunosenescence, characterized by diminished transcriptional plasticity and impaired NFκB and TLR pathways [48,49]. The convergence of obesity and aging in a state of “inflammaging” may further shift inflammatory activity toward metabolic tissues and senescent cells, rather than circulating immune cells. Thus, systemic cytokine alterations may not translate into transcriptional activation in PBMCs, highlighting a dissociation between systemic inflammation and immune-cell gene expression [50]. Regarding inflammatory markers, lower NFκB expression in neutrophils was observed in women with severe obesity compared with overweight women. NFκB activity is known to decrease following prolonged exposure to inflammatory stimuli, representing a form of “tolerance” or immune exhaustion [51]. Moreover, aging processes reduce NFκB pathway responsiveness in neutrophils, particularly in older individuals [48].

However, changes in GPx expression both in PBMCs and neutrophils were observed. GPx plays an important role in protecting mitochondrial and cytosolic compartments from H_2_O_2_-mediated oxidative damage [52]. GPx expression in PBMCs was significantly higher in women than in men across all obesity categories, supporting the concept that females maintain a more robust antioxidant defense. In this regard, estrogens have been shown to upregulate GPx and catalase expression, and to enhance global redox homeostasis in immune cells [53,54]. This gender-driven increase in GPx expression suggests that PBMCs from women exhibit greater resilience against mitochondrial ROS in obesity, potentially mitigating oxidative damage despite elevated systemic inflammation. However, these findings should be interpreted with caution, since RT-qPCR quantifies mRNA expression levels, which do not necessarily translate into corresponding increases in protein abundance or effective enzymatic activity. The observed increase in GPx transcripts may represent a compensatory or adaptive transcriptional response to oxidative stress rather than improved functional antioxidant protection. Additionally, the heterogeneous distribution of participants across groups may have influenced these results and should be considered when interpreting gender-related differences.

Additionally, women with severe obesity exhibited significantly higher expression of MitND5, a key subunit of mitochondrial complex I. These findings are in accordance with existing literature showing that women generally possess more efficient mitochondrial function in immune and metabolic tissues [55]. Estrogens promote mitochondrial biogenesis and oxidative phosphorylation by activating PGC-1α, NRF-1, and mitochondrial DNA replication machinery, supporting greater mitochondrial resilience in females under metabolic stress [55,56]. Such mechanisms likely explain the elevated MitND5 expression observed in PBMCs from women with higher obesity grades. In a similar way, women with overweight presented higher neutrophil GPx expression, but GPx levels progressively declined in those with moderate and severe obesity, while men did not exhibit any changes. In this sense, neutrophils are highly sensitive to metabolic stress, and their ability to maintain redox balance can be quickly exhausted under chronic inflammatory conditions [57].

Correlation analysis revealed that systemic cytokines were strongly interrelated, whereas their associations with immune-cell gene expression were generally weak. A strong correlation was observed between IL-15 plasma levels and TLR-2 expression in neutrophils. In this context, IL-15 has been proposed to modulate TLR-mediated responses and influence neutrophil activation, potentially acting as a counter-regulatory factor to limit excessive innate immune activation in the context of chronic metabolic inflammation [38,39]. Similarly, correlations between adipokines and gene expression were also weak. Leptin plasma levels were positively associated with MitND5 expression in PBMCs, while resistin correlated with COXIV in PBMCs and MTF-2 in neutrophils. These results suggest that adipokine dysregulation in obesity may influence mitochondrial turnover and bioenergetic adaptation in immune cells. It has been described that leptin can enhance mitochondrial oxidative metabolism in lymphocytes and monocytes, promoting both energy production and ROS generation [58], whereas resistin may modulate mitochondrial biogenesis and antioxidant defenses in immune cells through NFκB and PGC-1α-dependent pathways [59]. Collectively, these associations support the emerging concept that adipokines act as systemic modulators of immunometabolism, linking metabolic status to mitochondrial dynamics and functional adaptation in circulating immune cells.

This study has several limitations that should be acknowledged. First, the use of a convenience sample and the absence of a priori inferential sample size calculation may limit the generalizability of the findings. Second, the heterogeneous distribution of participants across obesity and gender categories may have influenced statistical power and effect estimates. Third, although information on comorbidities and pharmacological treatments was collected, these variables could not be fully controlled or used for stratification without substantially reducing sample size. Given that T2DM and commonly used medications such as statins and anti-inflammatory drugs can modulate inflammatory pathways, their potential confounding effects cannot be excluded. Finally, the reliance on gene expression data without parallel protein or functional measurements limits mechanistic interpretation.

## 4. Materials and Methods

### 4.1. Participants and Design

The study included a convenience sample of 134 participants (89 men and 45 women) aged 55–80 years, residing in the Balearic Islands. Participants were recruited from an ongoing observational study conducted in a clinical and community-based setting.

The wide age range was intentionally selected to include a population with a high prevalence of overweight, obesity, and obesity-related comorbidities, thereby enhancing the external validity of the findings and allowing the evaluation of obesity severity under real-world clinical conditions.

Participants were categorized according to body mass index (BMI) into three groups: overweight (25.0–29.9 kg/m^2^), moderate obesity (30.0–34.9 kg/m^2^), and severe obesity (35.0–40.0 kg/m^2^).

Participants with obesity-related comorbidities, including type 2 diabetes mellitus (T2DM), dyslipidemia, and hypertension, were explicitly eligible for inclusion provided that these conditions had been clinically diagnosed prior to recruitment. These comorbidities were therefore not considered incidental findings during the study.

Exclusion criteria included institutionalization, inability to complete questionnaires due to physical or mental conditions, alcoholism or drug dependence, and the use of investigational drugs within the previous year. Participants with T2DM were not excluded if they were under stable pharmacological treatment. Glycosylated hemoglobin (HbA1c) values were recorded to assess metabolic control at the time of inclusion.

The study protocols were approved by the Ethics Committee of Research of the Balearic Islands (ref. CEIC-IB1295/09PI and CEIC IB2251/14PI) in accordance with the Declaration of Helsinki. All participants were fully informed about the study procedures and provided written informed consent.

### 4.2. Anthropometric Assessment

Anthropometric measurements were performed by trained personnel following standardized procedures. Height was determined by using an anthropometer (Seca 214, SECA Deutschland, Hamburg, Germany). Participants were tested on their body weight and composition using a segmental body composition analyzer (Tanita BC-418, Tanita, Tokyo, Japan) while wearing light clothing and no shoes, and clothing was factored in with a weight allowance of 0.6 kg. BMI was calculated as weight (kg) divided by height squared (m^2^). Blood pressure was measured in the arm using a validated semi-automatic oscillometer (Omron HEM 705CP, Hoofddorp, The Netherlands), recording the highest diastolic value after three measurements following 5 min of seated rest, with a 1-min interval between measurements.

### 4.3. Plasma, Neutrophil, and PBMC Isolation

Venous blood samples were collected after a 12-h overnight fast into EDTA-containing tubes and centrifuged at 1700× *g* for 15 min at 4 °C to separate plasma, which was aliquoted and stored until analysis. PBMCs and neutrophil fractions were isolated from fresh blood following the protocol described by Bøyum (1964) [22]. PBMCs were obtained using Ficoll-Paque PLUS (GE Healthcare, Uppsala, Sweden) and subsequently washed with phosphate-buffered saline (PBS, pH 7.4). Neutrophils were purified using an adapted method involving erythrocyte lysis with ammonium chloride, followed by repeated PBS washes to ensure cell purity, as previously described [23].

### 4.4. Biochemical Parameters and Hemogram

Plasma glucose, triglycerides, total cholesterol, and HDL-c were determined using standard enzymatic assays. Hematological parameters and complete blood counts were measured with an automated flow cytometer (Technicon H2, VCS system, Bayer, Leverkusen, Germany).

### 4.5. RNA Extraction and Real-Time PCR

Total RNA was extracted from PBMCs and neutrophils using Tripure^®^ (Roche Diagnostics, Mannheim, Germany) according to previously established protocols [24]. One microgram of RNA from each sample was reverse-transcribed using Expand Reverse Transcriptase and oligo (dT) primers. The resulting cDNA was amplified using the LightCycler FastStart DNA MasterPLUS SYBR Green I kit (Roche Diagnostics, Germany). Gene expression analyses included COXIV, PGC-1α, MitND5, MTF-1 and MTF-2, with human 18S ribosomal RNA as the reference gene. The primers and amplification conditions used are listed in Table S6.

### 4.6. Cytokine Assays

Plasma cytokine concentrations were measured using MULTIPLEX ELISA kits (Diaclone, Besançon, France) according to the manufacturer’s instructions. Intra- and inter-assay coefficients of variation were 3.2% and 10.9% for TNFα and 4.4% and 9.1% for IL-6, respectively.

### 4.7. Statistical Analysis

Statistical analyses were conducted using SPSS v.25 (IBM, Chicago, IL, USA). Continuous variables are presented as mean ± standard deviation (SEM), with *p* < 0.05 considered statistically significant. Normality was assessed with the Kolmogorov–Smirnov test. A two-way analysis of variance (ANOVA) was applied to evaluate the effects of gender, obesity severity, and their interaction on the studied variables. When significant effects were detected, post hoc comparisons were performed using Bonferroni’s correction. Pearson correlation coefficients were calculated to assess associations between plasma cytokine and adipokine concentrations and gene expression levels in PBMCs and neutrophils.

## 5. Conclusions

In conclusion, this study evidenced that obesity severity and gender differentially modulate systemic inflammatory markers, hematological indices, and immune-related responses in older adults with overweight and obesity. While increasing obesity severity was associated with alterations in circulating cytokines, adipokines, and hematological parameters, particularly in a gender-dependent manner, gene expression changes in circulating immune cells were limited and selective. Notably, women exhibited greater variability in erythrocyte and leukocyte parameters and a higher expression of antioxidant and mitochondrial-related genes, whereas men with severe obesity showed a more pronounced increase in pro-inflammatory circulating mediators such as IL-6, IL-15, and IL-1rα. The weak associations observed between plasma inflammatory mediators and immune-cell gene expression suggest a dissociation between systemic inflammation and leukocyte transcriptional activity, potentially reflecting immune adaptation in the context of chronic metabolic stress and aging. Overall, these findings highlight the importance of considering gender as a key biological variable in obesity-related immunometabolic research.

## Figures and Tables

**Table 1:** Anthropometric and clinical characterization.

		Overweight 25.0 < BMI > 29.9	Moderate Obesity 30.0 < BMI > 34.9	Severe Obesity 35.0 < BMI > 40.0	ANOVA		
					G	O	GxO
**Patients**	**M**	32	39	18			
	**W**	13	28	24			
**BMI** **(Kg/m^2^)**	**M**	28.6 ± 1.04	32.3 ± 1.73 *	36.4 ± 1.98 *@	0.218	**0.001**	0.307
	**W**	28.4 ± 0.90	32.6 ± 1.34 *	37.3 ± 1.50 *@			
**Diastolic BP**	**M**	78.1 ± 8.4	79.7 ± 9.8	79.4 ± 8.1	0.380	0.534	0.764
	**W**	77.4 ± 6.9	79.3 ± 9.7	76.4 ± 9.2			
**Systolic BP**	**M**	135 ± 15	141 ± 18	139 ± 10	0.233	0.227	0.715
	**W**	134 ± 13	139 ± 14	132 ± 19			
**Glucose** **(mg/dL)**	**M**	116 ± 24	122 ± 43	119 ± 22	0.459	0.352	0.116
	**W**	129 ± 34	111 ± 23	113 ± 24			
**Cholesterol** **(mg/dL)**	**M**	167 ± 28	173 ± 43	173 ± 47	**0.002**	0.428	0.941
	**W**	184 ± 28	195 ± 37	195 ± 33			
**HDL-c** **(mg/dL)**	**M**	42.3 ± 8.2	40.7 ± 10.3 #	41.3 ± 11.4	**0.001**	0.269	0.107
	**W**	49.5 ± 10.4	55.3 ± 13.1	48.0 ± 10.2			
**LDL-c** **(mg/dL)**	**M**	97.1 ± 26.3	103 ± 37	101 ± 48 #	**0.021**	0.511	0.768
	**W**	107 ± 24	114 ± 34	121 ± 26			
**Triglycerides** **(mg/dL)**	**M**	147 ± 74	163 ± 139	156 ± 72	0.211	0.995	0.672
	**W**	146 ± 85	129 ± 63	133 ± 35			
**MDA** **(µM)**	**M**	5.07 ± 7.22	5.23 ± 3.83	7.46 ± 8.95	0.854	0.696	0.559
	**W**	3.98 ± 3.09	7.28 ± 6.77	5.46 ± 5.59			
**Polyphenols** **(mM)**	**M**	2.32 ± 1.58	1.52 ± 1.93	0.82 ±0.91	0.447	0.695	0.258
	**W**	1.84 ± 1.21	1.40 ± 1.37	3.02 ± 3.72			
**Nitrite** **(nM)**	**M**	256 ± 150	288 ± 195	335 ± 137	0.401	0.654	0.611
	**W**	240 ± 97	287 ± 201	252 ± 62			
**Nitrate** **(µM)**	**M**	77.2 ± 84.9	61.8 ± 62.3	52.5 ± 34.3	0.507	0.763	0.934
	**W**	57.4 ± 41.8	51.8 ± 79.0	49.1 ± 35.1			
**Nitrate/Nitrite** **(nM/nM)**	**M**	338 ± 503	213 ± 286	166 ± 127	0.753	0.614	0.826
	**W**	231 ± 284	225 ± 421	183 ± 151			

Results are the mean ± SEM. Statistical analysis: Two-way ANOVA, *p* < 0.05. G, means significant differences due to gender; O means significant differences between different obesity degrees; GxO, means interaction between two factors. # Indicates significant differences male and female; * indicates differences respect overweight; @ indicates differences between moderate obesity.

**Table 2:** Changes in Hematological parameters as consequence of obesity degree.

		Overweight 25.0 < BMI > 29.9	Moderate Obesity 30.0 < BMI > 34.9	Severe Obesity 35.0 < BMI > 40.0	ANOVA		
					G	O	GxO
Erythrocytes	**M**	4.77 ± 0.32 ^ab^	4.85 ± 0.50 ^a^	4.52 ± 0.44 ^ab^	0.083	0.630	**0.011**
	**W**	4.52 ± 0.27 ^ab^	4.54 ± 0.54 ^b^	4.71 ± 0.32 ^ab^			
Haemoglobin (g/L)	**M**	144 ± 7	139 ± 33	137 ± 16	0.561	0.238	0.882
	**W**	142 ± 2	139 ± 13	137 ± 12			
Glycosylated haemoglobin (%)	**M**	6.18 ± 0.85 ^ab^	6.36 ± 1.21 ^ab^	6.15 ± 0.71 ^ab^	0.524	**0.253**	**0.027**
	**W**	6.53 ± 0.95 ^a^	5.73 ± 0.79 ^b^	6.13 ± 0.63 ^ab^			
Haematocrit (%)	**M**	42.5 ± 2.3	43.2 ± 4.2 *	40.6 ± 4.4	0.345	**0.023**	0.514
	**W**	42.5 ± 1.5	41.6 ± 4.3	40.4 ± 4.1			
MCV (fL)	**M**	89.3 ± 3.8 ^ab^	89.3 ± 5.0 ^ab^	89.9 ± 6.6 ^ab^	0.212	**0.002**	**0.001**
	**W**	94.0 ± 3.0 ^a^	91.9 ± 3.9 ^a^	85.7 ± 6.1 ^b^			
Leukocytes	**M**	6.53 ± 1.56 #	6.41 ± 1.28	6.93 ± 1.41	**0.005**	**0.008**	0.063
	**W**	8.15 ± 2.43	6.50 ± 1.41 *	7.53 ± 1.73 @			
Neutrophils	**M**	3.79 ± 1.26 ^ab^	3.57 ± 1.00 ^a^	4.39 ± 1.06 ^ab^	0.170	**0.001**	**0.028**
	**W**	4.92 ± 2.19 ^b^	3.49 ± 0.94 ^a^	4.21 ± 1.18 ^ab^			
Lymphocytes	**M**	1.85 ± 050	1.98 ± 0.50	1.79 ± 0.57 #	**0.001**	0.966	0.264
	**W**	2.41 ± 0.49	2.23 ± 0.82	2.40 ± 0.76			
Monocytes	**M**	0.60 ± 0.18	0.56 ± 0.13	0.59 ± 0.13	0.302	0.226	0.812
	**W**	0.63 ± 0.14	0.57 ± 0.13	0.63 ± 0.17			
Eosinophils	**M**	0.26 ± 0.22 ^a^	0.25 ± 0.15 ^a^	0.21 ± 0.11 ^ab^	**0.025**	0.760	**0.039**
	**W**	0.14 ± 0.04 ^b^	0.17 ± 0.11 ^b^	0.24 ± 0.08 ^ab^			
Basophils	**M**	0.035 ± 0.02 #	0.043 ± 0.02	0.037 ± 0.03	**0.005**	0.924	0.377
	**W**	0.052 ± 0.03	0.047 ± 0.02	0.051 ± 0.03			
Platelets	**M**	221 ± 47	196 ± 43	201 ± 45	**0.001**	0.331	0.742
	**W**	243 ± 78	233 ± 67	240 ± 59			
NLR	**M**	2.26 ± 0.16	1.88 ± 0.14	2.57 ± 0.21 #@	**0.040**	**0.049**	0.218
	**W**	2.09 ± 0.25	1.79 ± 0.17	1.88 ± 0.18			
PLR	**M**	131 ± 8	104 ± 7	117 ± 10	0.224	0.532	0.162
	**W**	104 ± 12	111 ± 8	109 ± 9			
SII 10^9^ cell/L	**M**	512 ± 44	371 ± 40 *	506 ± 58	0.855	**0.007**	0.634
	**W**	564 ± 69	393 ± 47	455 ± 51			

Results are the mean ± SEM. Statistical analysis: Two-way ANOVA, *p* < 0.05. G, means significant differences due to gender; O means significant differences between different obesity degrees; GxO, means interaction between two factors. # Indicates significant differences male and female; * indicates differences respect overweight; @ indicates differences between moderate and severe obesity. When interaction exists between different statistical factors, different letters reveal significant differences.

**Table 3:** Plasma interleukin levels in men and women with overweight, moderate, or severe obesity.

Interleukin ng/mL		Overweight 25.0 < BMI > 29.9	Moderate Obesity 30.0 < BMI > 34.9	Severe Obesity 35.0 < BMI > 40.0	ANOVA		
					G	O	GxO
Ghrelin	**M**	314 ± 9 ^a^	298 ± 8 ^ab^	274 ± 12 ^b^	0.067	0.525	**0.011**
	**W**	259 ± 14 ^a^	289 ± 10 ^a^	290 ± 11 ^a^			
TNFα	**M**	3.39 ± 0.32	3.19 ± 0.29	3.31 ± 0.43	0.823	0.936	0.757
	**W**	3.12 ± 0.51	3.47 ± 0.35	3.50 ± 0.37			
OPG	**M**	23.0 ± 3.4	22.4 ± 3.0	26.0 ± 4.5	0.816	0.914	0.749
	**W**	23.4 ± 5.4	26.2 ± 3.7	24.1 ± 4.0			
IL-6	**M**	4.66 ± 1.15 ^a^	3.24 ± 1.05 ^a^	2.90 ± 1.54 ^a^	0.713	0.240	**0.034**
	**W**	0.75 ± 1.81 ^a^	2.20 ± 1.23 ^a^	6.62 ± 1.33 ^b^			
IL-10	**M**	0.67 ± 0.15	0.49 ± 0.13	0.58 ± 0.19	0.073	0.630	0.363
	**W**	0.12 ± 0.23	0.33 ± 0.16	0.53 ± 0.17			
IL1-rα	**M**	146 ± 87	201 ± 79	587 ± 116 *#@	**0.006**	**0.028**	0.191
	**W**	21.6 ± 136	78.5 ± 93	132 ± 100			
Resistin	**M**	8799 ± 3303	8566 ± 2897	7146 ± 4264	0.855	0.783	0.854
	**W**	6028 ± 5018	9992 ± 3419	6766 ± 3772			
IFN-γ	**M**	5.94 ± 0.45	5.27 ± 0.41	6.65 ± 0.60	0.629	0.188	0.648
	**W**	6.33 ± 0.71	5.87 ± 0.49	6.31 ± 0.52			
Leptin	**M**	6878 ± 3182	10,606 ± 2731	9441 ± 4500	0.658	0.191	0.683
	**W**	4203 ± 5077	14,020 ± 3765	12,962 ± 3765			
IL-15	**M**	5.09 ± 1.92	4.65 ± 1.74	14.2 ± 2.56 *#@	**0.044**	**0.053**	0.084
	**W**	3.75 ± 3.10	4.27 ± 2.05	4.51 ± 2.22			

Results are the mean ± SEM. Statistical analysis: Two-way ANOVA, *p* < 0.05. G, means significant differences due to gender; O means significant differences between different obesity degrees; GxO, means interaction between two factors. # Indicates significant differences male and female; * indicates differences respect overweight; @ indicates differences between moderate obesity. When interaction exists between different statistical factors, different letters reveal significant differences.

## Data Availability

Due to ethical and privacy restrictions related to the informed consent obtained from participants, the data supporting the findings of this study are not publicly available. Data may be made available from the corresponding author upon reasonable request and subject to ethical approval.

## Supplementary Materials

The following supporting information can be downloaded at: https://www.mdpi.com/article/10.3390/ijms27073314/s1.

## Abbreviations

The following abbreviations are used in this manuscript:

Akt	Protein kinase B
BMI	Body mass index
CAT	Catalase
COX-2	Cyclooxygenase 2
COXIV	Cytochrome c oxidase subunit IV
GPx	Glutathione peroxidase
HDL-c	High density protein cholesterol
IFN-γ	Gamma interferon
IKKβ	inhibitor of nuclear factor kappa-B kinase beta
IL	Interleukin
LDL-c	Low density protein cholesterol
MCP1	Monocyte chemoattractant protein-1
MCV	Corpuscular volume
MitND5	Mitochondrial NADH dehydrogenase subunit 5
MTF	Mitofusin
NFκB	Factor Nuclear kappa B
NLR	Neutrophil-to-lymphocyte ratio
OPG	Osteoprotegerin
PBMCs	Peripheral blood mononuclear cells
PI3K	phosphatidylinositol-3′-kinase
ROS	Reactive oxygen species
SII	The systemic immune-inflammatory index
TLR	Toll-like receptor
TNFα	Tumour necrosis factor α
