# Supplementary material for: Sex-Specific Effects of Obesity Severity on Circulating Inflammatory Mediators and Immune Cell Gene Expression
Source: Int J Mol Sci. 2026 Apr 7;27(7):3314. doi: 10.3390/ijms27073314 (PMC13072803; doi:10.3390/ijms27073314)
Supplement: Supplementary file 1 [file ijms-27-03314-s001.zip › Table S6.pdf]

Table S6. Primers sequences and Real-Time PCR conditions.

Gen	Primer	Annealing Temperature
**COXIV**	**Fw:** 5′-AGAAGCACTATGTGTACGGCCC-3′<br>**Rv:** 5′-GGTTCACCTTCATGTCCAGCAT-3′	**63 °C**
**MitND5**	**Fw:** 5′-CGGCTGAGAGGGCGTAGG-3′<br>**Rv:** 5′-GATGAAACCGATATCCGGCCGA-3′	**63 °C**
**MTF-1**	**Fw:** 5′-TGTTTTGGTCGCAAACTCTG-3′<br>**Rv:** 5′-CTGTCTGCGTACGTCTTCCA-3′	**60 °C**
**MTF-2**	**Fw:** 5′-ATGCATCCCCACTTAAGCAC-3′<br>**Rv:** 5′-CCAGAGGGCAGAACTTTGTC-3′	**60 °C**
**Rib**	**Fw:** 5′-ATGTGAAGTCACTGTGCCAG-3′<br>**Rev:** 5′-GTGTAATCCGTCTCCACAGA-3′	**60 °C**
**NFκB**	**Fw:** 5′- AAACACTGTGAGGATGGGATCTG-3′<br>**Rv:**5′- CGAAGCCGACCACCATGT-3′	**60 °C**
**TNFα**	**Fw:** 5'-CCCAGGCAGTCAGATCATCTTCTCGGAA-3'<br>**Rv:** 5'-CTGGTTATCTCTCAGCTCCACGCCATT- 3'	**63 °C**
**IL-10**	**Fw:** 5′-AGAACCTGAAGACCCTCAGGC -3′<br>**Rv:** 5′-CCACGGCCTTGCTCTTGTT -3′	**58 °C**
**IL-1β**	**Fw:** 5'-GGACAGGATATGGAGCAACA -3'<br>**Rv:** 5'-GGCAGACTCAAATTCCAGCT -3'	**58 °C**
**TLR-4**	**Fw:** 5′-GGTCACCTTTTCTTGATTCCA-3′<br>**Rv:** 5′- TCAGAGGTCCATCAAACATCAC-3′	**55 °C**
**TLR-2**	**Fw:** 5′-GGGTTGGAAGCACTGGACAAT-3′<br>**Rv:** 5′-TTCTTCCTTGGAGAGGCTGA-3′	**55 °C**
**COX-2**	**Fw:** 5′-TTGCCTGGCAGGGTTGCTGGTGGTA-3′<br>**Rv:** 5′-CATCTGCCTGCTCTGGTCAATGGAA-3′	**63 °C**
**GPx**	**Fw:** 5′-TTCCCGTGCAACCAGTTTG-3′<br>**Rv:** 5′-TTCACCTCGCACTTCTCGAA-3′	**63 °C**

Fw: Forward; Rv: Reverse, COXIV, cytochrome c oxidase subunit IV; MitND5, Mitochondrial NADH Dehydrogenase Subunit 5; MTF 1, Mitofusin 1; MTF2, mitofusin 2, Rib, ribosomal 18S; NFkB, nuclear factor κ Beta; TNFα, Tumour Necrosis factor α; IL-10, Interleukin 10; IL-1β, Interleukin 1β; TLR-2, toll like receptor 2; TLR-4, toll like receptor 4; COX-2, Cyclooxygenase 2; GPx, Glutathione peroxidase.
