# Supplementary material for: Sex-Specific Effects of Obesity Severity on Circulating Inflammatory Mediators and Immune Cell Gene Expression
Source: Int J Mol Sci. 2026 Apr 7;27(7):3314. doi: 10.3390/ijms27073314 (PMC13072803; doi:10.3390/ijms27073314)
Supplement: Supplementary file 1 [file ijms-27-03314-s001.zip › Table S1.pdf]

Table S1. Statistical parameters, including degrees of freedom and F-values from table 1.

		F-value	Degrees of freedom
BMI (Kg/m^2)	**O**	311.01	1
	**G**	1.533	2
	**OxG**	1.190	2
Diastolic BP (mmHg)	**O**	0.630	1
	**G**	0.774	2
	**OxG**	0.270	2
Systolic BP (mmHg)	**O**	1.498	1
	**G**	1.437	2
	**OxG**	0.336	2
Glucose (mg/dL)	**O**	1.051	1
	**G**	0.550	2
	**OxG**	2.185	2
Cholesterol (mg/dL)	**O**	0.853	1
	**G**	10.089	2
	**OxG**	0.061	2
HDL-c (mg/dL)	**O**	1.326	1
	**G**	27.091	2
	**OxG**	2.273	2
LDL-c (mg/dL)	**O**	0.675	1
	**G**	0.265	2
	**OxG**	5.433	2
Triglycerides (mg/dL)	**O**	0.005	1
	**G**	1.579	2
	**OxG**	0.398	2
MDA (µM)	**O**	0.365	1
	**G**	0.034	2
	**OxG**	0.588	2
Polyphenols (mM)	**O**	0.366	1
	**G**	0.587	2
	**OxG**	1.389	2
Nitrite (nM)	**O**	0.427	1
	**G**	0.714	2
	**OxG**	0.496	2
Nitrate (µM)	**O**	0.272	1
	**G**	0.446	2
	**OxG**	0.068	2
Nitrate/Nitrite (nM/nM)	**O**	0.490	1
	**G**	0.100	2
	**OxG**	0.192	2
