# Supplementary material for: Sex-Specific Effects of Obesity Severity on Circulating Inflammatory Mediators and Immune Cell Gene Expression
Source: Int J Mol Sci. 2026 Apr 7;27(7):3314. doi: 10.3390/ijms27073314 (PMC13072803; doi:10.3390/ijms27073314)
Supplement: Supplementary file 1 [file ijms-27-03314-s001.zip › Table S2.pdf]

Table S2. Statistical parameters, including degrees of freedom and F-values from table 2.

		F-value	Degrees of freedom
Erythrocytes	**O**	0.463	1
	**G**	3.037	2
	**OxG**	4.363	2
Haemoglobin (g/L)	**O**	1.455	1
	**G**	0.340	2
	**OxG**	0.126	2
Glycosylated haemoglobin (%)	**O**	1.386	1
	**G**	0.408	2
	**OxG**	3.703	2
Haematocrit (%)	**O**	3.893	1
	**G**	0.897	2
	**OxG**	0.688	2
MCV (fL)	**O**	6.613	1
	**G**	1.571	2
	**OxG**	9.234	2
Leukocytes	**O**	4.959	1
	**G**	8.099	2
	**OxG**	2.818	2
Neutrophils	**O**	7.589	1
	**G**	1.904	2
	**OxG**	3.668	2
Lymphocytes	**O**	0.035	1
	**G**	19.742	2
	**OxG**	1.344	2
Monocytes	**O**	1.502	1
	**G**	1.074	2
	**OxG**	0.208	2
Eosinophils	**O**	0.275	1
	**G**	5.100	2
	**OxG**	3.305	2
Basophils	**O**	0.079	1
	**G**	8.05	2
	**OxG**	0.983	2
Platelets	**O**	1.114	1
	**G**	11.900	2
	**OxG**	0.299	2
NLR	**O**	2.300	1
	**G**	0.432	2
	**OxG**	1.839	2
PLR	**O**	3.460	1
	**G**	0.681	2
	**OxG**	0.343	2
SII 10^9 cell/L	**O**	3.825	1
	**G**	3.142	2
	**OxG**	1.809	2
