# Supplementary material for: Sex-Specific Effects of Obesity Severity on Circulating Inflammatory Mediators and Immune Cell Gene Expression
Source: Int J Mol Sci. 2026 Apr 7;27(7):3314. doi: 10.3390/ijms27073314 (PMC13072803; doi:10.3390/ijms27073314)
Supplement: Supplementary file 1 [file ijms-27-03314-s001.zip › Table S3.pdf]

Table S3. Statistical parameters, including degrees of freedom and F-values from table 3.

Interleukin		F-value	Degrees of freedom
Ghrelin	**O**	0.258	2
	**G**	3.569	1
	**OxG**	4.773	2
TNFα	**O**	0.104	2
	**G**	0.087	1
	**OxG**	0.327	2
OPG	**O**	0.097	2
	**G**	0.03	1
	**OxG**	0.059	2
IL-6	**O**	1.567	2
	**G**	0.042	1
	**OxG**	3.949	2
IL-10	**O**	0.230	2
	**G**	3.702	1
	**OxG**	1.317	2
IL1-rα	**O**	3.684	2
	**G**	6.991	1
	**OxG**	1,450	2
Resistin	**O**	0.470	2
	**G**	0.034	1
	**OxG**	0.151	2
IFN- γ	**O**	1.594	2
	**G**	0.291	1
	**OxG**	0.445	2
Leptin	**O**	1.193	2
	**G**	0.014	1
	**OxG**	0.419	2
IL-15	**O**	2.582	2
	**G**	3.008	1
	**OxG**	1.869	2

		F-value	Degrees of freedom
Erythrocytes	**O**	0.463	1
	**G**	3.037	2
	**OxG**	4.363	2
Haemoglobin (g/L)	**O**	1.455	1
	**G**	0.340	2
	**OxG**	0.126	2
Glycosylated haemoglobin (%)	**O**	1.386	1
	**G**	0.408	2
	**OxG**	3.703	2
Haematocrit (%)	**O**	3.893	1
	**G**	0.897	2
	**OxG**	0.688	2
MCV (fL)	**O**	6.613	1
	**G**	1.571	2
	**OxG**	9.234	2
Leukocytes	**O**	4.959	1
	**G**	8.099	2
	**OxG**	2.818	2
Neutrophils	**O**	7.589	1
	**G**	1.904	2
	**OxG**	3.668	2
Lymphocytes	**O**	0.035	1
	**G**	19.742	2
	**OxG**	1.344	2
Monocytes	**O**	1.502	1
	**G**	1.074	2
	**OxG**	0.208	2
Eosinophils	**O**	0.275	1
	**G**	5.100	2
	**OxG**	3.305	2
Basophils	**O**	0.079	1
	**G**	8.05	2
	**OxG**	0.983	2
Platelets	**O**	1.114	1
	**G**	11.900	2
	**OxG**	0.299	2
NLR	**O**	2.300	1
	**G**	0.432	2
	**OxG**	1.839	2
PLR	**O**	3.460	1
	**G**	0.681	2
	**OxG**	0.343	2
SII 10^9 cell/L	**O**	3.825	1
	**G**	3.142	2
	**OxG**	1.809	2
