# Supplementary material for: Sex-Specific Effects of Obesity Severity on Circulating Inflammatory Mediators and Immune Cell Gene Expression
Source: Int J Mol Sci. 2026 Apr 7;27(7):3314. doi: 10.3390/ijms27073314 (PMC13072803; doi:10.3390/ijms27073314)
Supplement: Supplementary file 1 [file ijms-27-03314-s001.zip › Table S4.pdf]

Table S4. PBMCs And Neutrophils Gene Expression

Gene expresión		Overweight 25.0<BMI>29.9	Moderate Obesity 30.0<BMI>34.9	Severe Obesity 35.0<BMI>40.0	ANOVA S	ANOVA O	ANOVA SxO
PBMCs							
TNF-α	M	1.00 ± 0.24	0.61 ± 0.25	0.93 ± 0.32	0.340	0.935	0.546
(AU)	W	0.45 ± 0.33	0.73 ± 0.38	0.64 ± 0.30			
IL-1β	M	1.00 ± 0.52	0.93 ± 0.58	2.47 ± 0.66	0.384	0.221	0.575
(AU)	W	1.87 ± 0.75	1.70 ± 0.61	2.15 ± 0.59			
IL-10	M	1.00 ± 0.43	0.40 ± 0.30	0.25 ± 0.37	0.822	0.306	0.354
(AU)	W	0.31 ± 0.58	1.02 ± 0.53	0.73 ± 0.33			
TLR-2	M	1.00 ± 0.22	0.69 ± 0.20	0.56 ± 0.23	0.429	0.392	0.846
(AU)	W	0.99 ± 0.27	0.94 ± 0.26	0.78 ± 0.23			
TLR-4	M	1.00 ± 0.24	0.65 ± 0.21	0,71 ± 0.24	0.839	0.283	0.963
(AU)	W	1.08± 0.29	0.72 ± 0.26	0.67 ± 0.24			
COXIV	M	1.00 ± 0.43	0.89 ± 0.37	1.94 ± 0.44	0.481	0.742	0.124
(AU)	W	1.05 ± 0.45	1.31 ± 0.43	0.74 ± 0.41			
MTF-1	M	1.00 ± 0.47	1.01 ± 0.45	1.24 ± 0.52	0.598	0.946	0.765
(AU)	W	1.15 ± 0.64	0.83 ± 0.56	0.58 ± 0.58			
MTF-2	M	1.00 ± 1.15	2.71 ± 0.99	0.54 ± 1.22	0.635	0.595	0.552
(AU)	W	1.67 ± 1.66	0.67 ± 1.37	0.41 ± 1.23			
MitND5	M	1.00 ± 0.36	0.86 ± 0.31	0.70 ± 0.37#	**0.018**	0.939	0.545
(AU)	W	1.29 ± 0.46	1.63 ± 0.38	1.86 ± 0.37			
NFκB	M	1.00 ± 0.25	1.12 ± 0.22	0.83 ± 0.26	0.543	0.722	0.442
(AU)	W	1.34 ± 0.29	0.87 ± 0.29	1.13 ± 0.25			
COX-2	M	1.00 ± 0.32	1.13 ± 0.30	0.58 ± 0.38	0.588	0.635	0.614
(AU)	W	1.29 ± 0.42	0.90 ± 0.38	1.01 ± 0.35			
GPx	M	1.00 ± 1.11	0.71 ± 0.93#	1.10 ± 1.11	**0.030**	0.478	0.283
(AU)	W	2.74 ± 1.36	4.53 ± 1.11	1.58 ± 1.05			
Neutrophils							
TNF-α	M	1.00 ± 2.65	3.43 ± 2.91	1.37 ± 4.12	0.366	0.321	0.747
(AU)	W	2.13 ± 4.39	9.27 ± 3.71	2.63 ± 4.12			
IL-1β	M	1.00 ± 0.42	0.61 ± 0.51	0.26 ± 0.88	0.066	0.051	0.206
(AU)	W	3.32 ± 0.95	0.57 ± 0.62	1.21 ± 0.70			
TLR-2	M	1.00 ± 0.59	0.20 ± 0.62	0.82 ± 0.81	0.105	0.566	0.094
(AU)	W	0.54 ± 0.94	2.91 ± 0.75	1.64 ± 0.87			
TLR-4	M	1.00 ± 0.60	0.36 ± 0.62	0.18 ± 0.85	0.873	0.629	0.376
(AU)	W	0.10 ± 0.92	1.54 ± 0.80	0.21 ± 0.88			
MTF-1	M	1.00 ± 1.69	0.02 ± 1.63	4.20 ± 3.21	0.228	0.102	0.496
(AU)	W	0.21 ± 2.74	0.02 ± 2.20	1.21 ± 2.34			
MTF-2	M	1.00 ± 0.18	0.38 ± 0.21	0.54 ± 0.30	0.639	0.480	0.462
(AU)	W	0.52 ± 0.33	0.52 ± 0.29	0.56 ± 0.30			
COXIV	M	1.00 ± 5.95	12.5 ± 6.39	1.15 ± 8.40	0.648	0.532	0.552
(AU)	W	13.2 ± 8.90	8.85 ± 8.01	1.65 ± 9.81			
MitDN5	M	1.00 ± 0.42	1.12 ± 0.45	0.91 ± 0.61	0.580	0.393	0.401
(AU)	W	1.61 ± 0.64	0.52 ± 0.53	0.15 ± 0.63			
NFκB	M	1.00 ± 0.19	0.60 ± 0.21	0.51 ± 0.21	0.147	**0.041**	0.877
(AU)	W	0.82 ± 0.28	0.35 ± 0.22	0.07 ± 0.30			

COX-2 (AU)	M	1.00 ± 0.40	0.95 ± 0.43	1.16 ± 0.60	0.239	0.907	0.745
	W	0.78 ± 0.64	0.63 ± 0.51	0.10 ± 0.67			
GPx (AU)	M	1.00 ± 1.15[a]	2.34 ± 1.06[a]	1.15 ± 1.91[a]	0.441	0.190	**0.026**
	W	5.15 ± 2.21[b]	0.61 ± 1.56[a]	1.47 ± 1.80[a]			
CAT (AU)	M	1.00 ± 0.38	0.81 ± 0.43	0.34 ± 0.68	0.919	0.411	0.667
	W	0.58 ± 0.83	1.40 ± 0.56	0.32 ± 0.61			

Results are the mean ± SEM. Statistical analysis: Two-way ANOVA, $p < 0.05$. G, means significant differences due to gender; O means significant differences between different obesity degrees; GxO, means interaction between two factors. # Indicates significant differences male and female; * indicates differences respect overweight; @ indicates differences between moderate obesity. When interaction exists between different statistical factors, different letters reveal significant differences.
