# Supplementary material for: Sex-Specific Effects of Obesity Severity on Circulating Inflammatory Mediators and Immune Cell Gene Expression
Source: Int J Mol Sci. 2026 Apr 7;27(7):3314. doi: 10.3390/ijms27073314 (PMC13072803; doi:10.3390/ijms27073314)
Supplement: Supplementary file 1 [file ijms-27-03314-s001.zip › Table S5.pdf]

Table S5. Statistical parameters, including degrees of freedom and F-values from table S4.

PBMCs gene Expression		F-value	Degrees of freedom
TNF-α	**O**	0.068	2
	**G**	0.921	1
	**OxG**	0.609	2
IL-1β	**O**	1.535	2
	**G**	0.765	1
	**OxG**	0.557	2
TLR-2	**O**	0.943	2
	**G**	0.629	1
	**OxG**	0.168	2
TLR-4	**O**	1.273	2
	**G**	0.042	1
	**OxG**	0.037	2
COXIV	**O**	0.299	2
	**G**	0.500	1
	**OxG**	2.124	2
MTF-1	**O**	0.055	2
	**G**	0.279	1
	**OxG**	0.269	2
MTF-2	**O**	0.521	2
	**G**	0.226	1
	**OxG**	0.597	2
MitND5	**O**	0.063	2
	**G**	5.728	1
	**OxG**	0.609	2
NFκB	**O**	0.326	2
	**G**	0.372	1
	**OxG**	0.820	2
COX-2	**O**	0.456	2
	**G**	0.295	1
	**OxG**	0.490	2
GPx	**O**	0.741	2
	**G**	4.830	1
	**OxG**	1.275	2

Neutrophils gene Expression

TNF-α	**O**	1.145	2
	**G**	0.824	1
	**OxG**	0.292	2
IL-1β	**O**	3.086	2
	**G**	3.472	1
	**OxG**	1.611	2

IL-10	**O**	0.097	2
	**G**	0.03	1
	**OxG**	0.059	2
TLR-2	**O**	0.571	2
	**G**	2.658	1
	**OxG**	2.401	2
TLR-4	**O**	0.465	2
	**G**	0.026	1
	**OxG**	0.985	2
COXIV	**O**	0.633	2
	**G**	0.209	1
	**OxG**	0.598	2
MTF-1	**O**	1.467	2
	**G**	4.585	1
	**OxG**	0.704	2
MTF-2	**O**	0.740	2
	**G**	0.222	1
	**OxG**	0.77	2
MitND5	**O**	0.939	2
	**G**	0.308	1
	**OxG**	0.919	2
NFκB	**O**	3.303	2
	**G**	2.139	1
	**OxG**	0.131	2
COX-2	**O**	0.098	2
	**G**	1.400	1
	**OxG**	0.295	2
GPx	**O**	1.273	2
	**G**	0.866	1
	**OxG**	3.406	2
CAT	**O**	0.900	2
	**G**	0.010	1
	**OxG**	0.407	2
